# Epigenetic effects of the natural flavonolignan silibinin on colon adenocarcinoma cells and their derived metastatic cells

**DOI:** 10.3892/ol.2013.1190

**Published:** 2013-02-12

**Authors:** HENRIETTE KAUNTZ, SOUAD BOUSSEROUEL, FRANCINE GOSSÉ, FRANCIS RAUL

**Affiliations:** 1 Department of Nutritional Cancer Prevention, University of Strasbourg, Unit EA 4438, Faculty of Medicine;; 2 IRCAD-EITS, F-67000 Strasbourg, France

**Keywords:** colorectal cancer, silibinin, HDAC inhibitors, DNMT inhibitors, combination therapy

## Abstract

Epigenetic modifications are important in tumorigenesis. The most frequent epigenetic phenomena in cancer are histone deacetylation and DNA hypermethylation, which lead to gene silencing, particularly of tumor suppressor genes. However, monotherapies with histone deacetylase (HDAC) or DNA methyltransferase (DNMT) inhibitors lack efficacy, hence there is a need to enhance their anticancer action in a safe and effective combination therapy. The present study investigated the epigenetic effects of the natural flavonolignan silibinin in a model of colon cancer progression, the primary adenocarcinoma cells SW480 and their derived metastatic cells SW620. Silibinin did not change the activity of HDACs, but it was able to significantly inhibit DNMT activity in both SW480 and SW620 cells. The clinically used HDAC inhibitor, suberoylanilide hydroxamic acid (SAHA), and the broad spectrum HDAC inhibitor, trichostatin A (TSA), combined with silibinin demonstrated synergistic effects on cell death induction, may be related to its DNMT inhibition properties. The present data suggest that treatments combining silibinin and HDAC inhibitors may represent a promising approach, given the non-toxic nature of silibinin and the fact that HDAC inhibitors selectively target cancer cells.

## Introduction

Epigenetic modifications affect gene expression. Tumors often show an aberrant epigenetic modification pattern, including histone deacetylation and DNA hypermethylation, which leads to the suppression of gene expression (1). Colorectal cancer (CRC) in particular results not only from an accumulation of genetic changes, but also epigenetic changes, which occur in the course of the transformation of normal epithelium into adenocarcinomas.

Histone deacetylases (HDACs) catalyze the removal of acetyl groups, thereby stimulating chromatin condensation, which promotes transcriptional repression, including the shutdown of tumor suppressor gene expression (2). HDACs are also able to target numerous non-histone proteins, such as p53, even accounting for a majority of HDAC substrates (3). Overexpression of HDACs and epigenetic silencing has been frequently observed in CRC (4,5). Amongst others, these studies show that the perturbation of the balance between acetylation and deacetylation plays an important role in neoplastic transformation. As epigenetic changes are reversible, HDACs constitute promising targets for pharmacological inhibition in CRC (3).

By mechanisms not yet fully elucidated, HDAC inhibitors are able to induce cell-cycle arrest, promote differentiation and selectively stimulate apoptosis in transformed cells via the extrinsic and/or intrinsic apoptotic pathway (6). Furthermore, they synergistically enhance the anticancer activity of chemotherapeutic drugs, particularly those that are pro-apoptotic, by shifting the balance between pro- and anti-apoptotic proteins.

Examples of HDAC inhibitors include short-chain fatty acids, hydroxamic acids, benzamides and cyclic tetrapeptides (2). The broad spectrum HDAC inhibitor SAHA (also known as Zolinza^®^ or Vorinostat) was the first HDAC inhibitor to have successfully completed clinical trials and is used for the treatment of cutaneous T-cell lymphoma (7). Numerous HDAC inhibitors are currently undergoing clinical trials (2). TSA is another hydroxamic acid and broad spectrum HDAC inhibitor which targets class I, II and IV HDACs in the same way as SAHA (3).

DNA hypermethylation is another frequent phenomenon in cancer that silences many genes for cell cycle regulation, receptors and apoptosis by DNA methylation of CpG islands in their promoter region (8). A proportion of proximal colon tumors (30–40%), and distal and rectal tumors (3–12%), exhibit a CpG island methylator phenotype, where numerous CpG islands are methylated and various tumor suppressor genes are inactivated (9).

DNA methyltransferase (DNMT) inhibitors are able to induce DNA demethylation and thus the reactivation of epigenetically silenced genes. At present, there are two FDA-approved drugs with DNMT inhibitory activity: 5-azacytidine and decitabine, both of which are used in the treatment of myelodysplastic syndrome and myeloid leukemias (3). Global reduction of DNA methylation has been demonstrated to have anticancer effects in intestinal tumorigenesis (10).

Histone deacetylation has been revealed to act synergistically with DNA methylation in the epigenetic silencing of cancer genes (11). Therefore combinations of DNMT and HDAC inhibitors may have potential for further clinical trials (10).

Natural substances have been found to act on epigenetic signaling; green tea polyphenols demonstrated similar effects to TSA in prostate cancer cells by inducing cell cycle arrest and apoptosis (12). These polyphenols were able to inhibit HDACs and induce their proteasomal degradation. Furthermore, the tea polyphenol (-)-epigallocatechin-3-gallate was also able to inhibit DNMT activity and reactivate methylation-silenced genes in CRC cells (13). In addition, apple polyphenols reduced DNA methylation by inhibition of DNMT in CRC cells (14). Moreover, soy isoflavones reversed DNA hypermethylation and reactivated silenced genes in esophageal squamous cell carcinoma cells (15), and their anticancer activity was enhanced when used in combination with HDAC inhibitors (15). The polyphenol curcumin has also been shown to inhibit DNMT activity (16) and to act synergistically with TSA in inducing cell death (17).

The flavonolignan silibinin, which is the main pharmacologically active component of the milk thistle plant (*Silybum marianum),* has been shown to increase acetylation of histones in hepatic cancer *in vitro* and *in vivo*. Silibinin increased acetylation of histone H3 and H4 *in vitro* in HuH7 cells (18), and *in vivo* in HuH7 xenografts in nude mice (19). In non-small cell lung cancer, silibinin inhibited HDAC activity and decreased HDAC levels (20).

However, no studies have yet to fully describe the effect of silibinin on DNMT activity. Previously, we demonstrated that silibinin exerted anti-proliferative and pro-apoptotic effects in the primary adenocarcinoma SW480 cells and in their metastatic derivatives (SW620 cells) (21). In the present study, we aimed to investigate whether silibinin modified HDAC and DNMT activity in this model of CRC progression.

## Material and methods

### Cell culture and treatment

SW480 and SW620 cells were purchased from the European Collection of Animal Cell Culture (ECACC, Salisbury, UK). The cells were cultured in 75 cm^2^ Falcon flasks in Dulbecco’s modified Eagle’s medium (DMEM) containing 25 mM glucose and supplemented with 10% heat-inactivated (56°C) horse serum, 100 U/ml penicillin, 100 *μ*g/ml streptomycin and 1% non-essential amino acids (Invitrogen Life Technologies, Lyon, France). Cells were maintained at 37°C in a humidified atmosphere with 5% CO_2_, and subcultured following trypsinization (0.5% trypsin/2.6 mM ethylendiamine tetraacetic acid). For all experiments, cells were seeded at 1×10^6^ cells in culture dishes (10 cm internal diameter). The culture medium used was DMEM supplemented with 3% heat-inactivated horse serum and 100 U/ml penicillin, 100 *μ*g/ml streptomycin and 1% non-essential amino acids. Additionally, 5 *μ*g/ml transferrin, 5 ng/ml selenium and 10 *μ*g/ml insulin were added to compensate for the lower serum concentration. The culture medium was replaced every 48 h. Cells were exposed to silibinin (Sigma-Aldrich Chemie GmbH, Steinheim, Germany) 24 h after seeding. Silibinin was dissolved in dimethylsulf-oxide (DMSO; Sigma-Aldrich Chemie GmbH) and used at a final concentration of 300 *μ*M. The final concentration of DMSO in the culture medium did not exceed 0.1%. The HDAC inhibitors, TSA (0.1 *μ*M) or SAHA (1 *μ*M) (Sigma-Aldrich Chemie GmbH), were added 1 h prior to silibinin treatment.

### Histone deacet ylase (HDAC) and DNA methyltransferase (DNMT) activity

To determine the activity/inhibition of the HDACs/DNMTs in the nuclear samples, the colorimetric EpiQuik™ HDAC Activity/Inhibition Assay kit (Epigentek-Euromedex, Strasbourg, France) and the colorimetric EpiQuik™ DNMT Activity/Inhibition Assay Ultra kit (Epigentek-Euromedex) were used. Cells were harvested by scraping, and nuclear extracts of the cells were prepared using the EpiQuik™ Nuclear Extraction kit (Epigentek-Euromedex). The protein content of the nuclear extracts was determined by the Lowry assay, and any nuclear extracts not immediately used were stored at −80°C.

To measure the HDAC activity, the same quantity of nuclear extract was incubated with acetylated histone substrate in a 96-well plate, and the quantity of remaining un-deacetylated histone that is inversely proportional to HDAC enzyme activity in the nuclear sample was colorimetrically quantified through an ELISA-like reaction at a wavelength of 450 nm. The activity of HDAC enzymes was inversely proportional to the OD.

To measure the DNMT activity, nuclear extracts were incubated on a microplate stably coated with a universal DNMT substrate. DNMT enzymes from the nuclear sample methylate the DNA substrate during incubation, and then the quantity of methylated DNA that is proportional to enzyme activity was colorimetrically quantified through an ELISA-like reaction at a wavelength of 450 nm. The activity of DNMT enzymes was proportional to the OD.

### Flow cytometric analysis of the sub-G0/G1 cell population

The sub-G0/G1 cell population (hypodiploid cells: dying and dead cells) was analyzed by labeling cells with propidium iodide. Cells were harvested by trypsinization after 24, 48 and 72 h of treatment and washed with phosphate-buffered saline (PBS; 0.1 M; pH 7.4). Cells were fixed in 70% ethanol at −20°C for ≥30 min, washed twice with PBS and re-suspended in 200 *μ*l PBS containing 0.25 mg/ml RNase A and 0.1 mg/ml propidium iodide (Sigma-Aldrich Chemie GmbH). Following incubation in the dark at 37°C for 30 min, the fluorescence of 10,000 cells/sample was analyzed by flow cytometry, and histograms were analyzed using the CellQuest software (FACScan, BD Biosciences, Belgium).

### Statistical analysis

All experiments were performed ≥3 times. Data are presented as mean ± standard error. Statistical differences between the control and treated groups were evaluated using the Student’s t-test or the Student-Newman-Keuls multiple comparison test. P<0.05 was considered to indicate a statistically significant difference between groups.

## Results

### Effects of silibinin on HDAC and DNMT activity

To establish whether silibinin induced epigenetic modifications in SW480 and SW620 cells, we determined HDAC and DNMT activity in nuclear extracts of silibinin-treated cells with the aid of the colorimetric EpiQuik HDAC Activity/Inhibition Assay kit and the EpiQuik DNMT Activity/Inhibition Assay Ultra kit.

**Figure 1:**
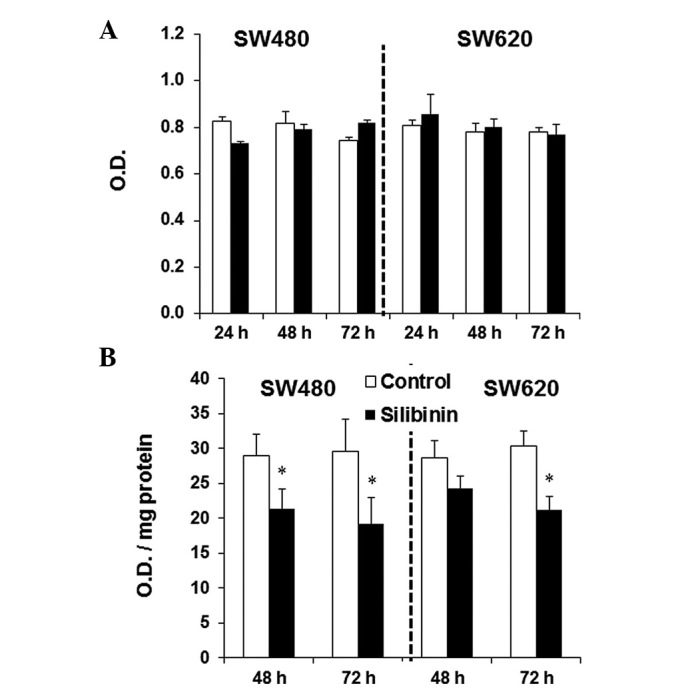
Effect of silibinin on HDAC and DNMT activity. SW480 and SW620 cells were treated with DMSO 0.1% ± silibinin (300 *μ*M) for 24, 48 or 72 h. Nuclear extracts were prepared, and HDAC (A) or DNMT (B) activity was measured by colorimetric methods as detailed in Materials and methods. Data are presented as the mean ± standard error of three separate experiments. Data are expressed as the OD for equal amounts of protein (A) or as OD/mg protein (B). For each cell line, silibinin treatment vs. non-treated control: ^*^P<0.05.

HDAC activity in SW480 and SW620 cells was not changed by silibinin treatment (Fig. 1A). In contrast, a reduction in DNMT activity was observed in silibinin-treated SW480 and SW620 cells after 48 h of treatment with silibinin (Fig. 1B). However, this reduction only became significant following 72 h of treatment in SW620 cells.

### Silibinin and HDAC inhibitors induce synergistic cell death

Because synergy between DNA demethylation and HDAC inhibition has been demonstrated in the re-expression of genes silenced in cancer (11), we investigated the synergistic effect of silibinin, which inhibits DNMT as shown, in combination with HDAC inhibitors on cell death.

**Figure 2:**
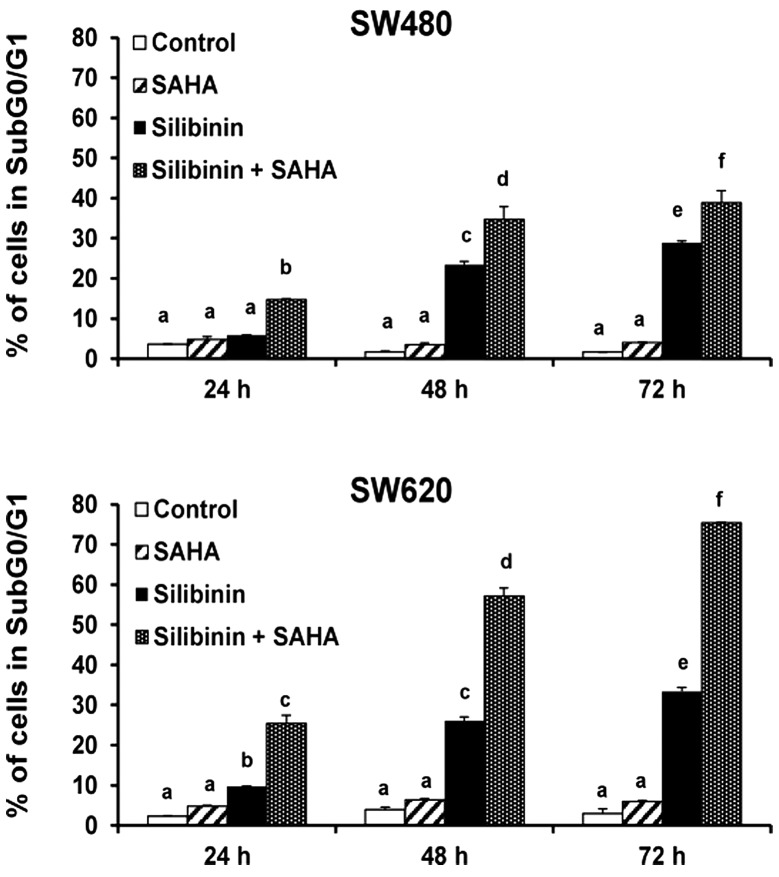
Cell death induced by silibinin and SAHA. SW480 and SW620 cells were treated with DMSO 0.1% ± silibinin (300 *μ*M) ± SAHA (1 *μ*M) for 24, 48 or 72 h. At each time point, SW480 and SW620 cells were harvested and stained with propidium iodide for the measurement of hypodiploid bodies and analyzed by flow cytometry as detailed in Materials and methods. Data are presented as the mean ± standard error of three separate experiments. For each cell line, columns that do not share the same superscript differ significantly: ^*^P<0.05.

**Figure 3:**
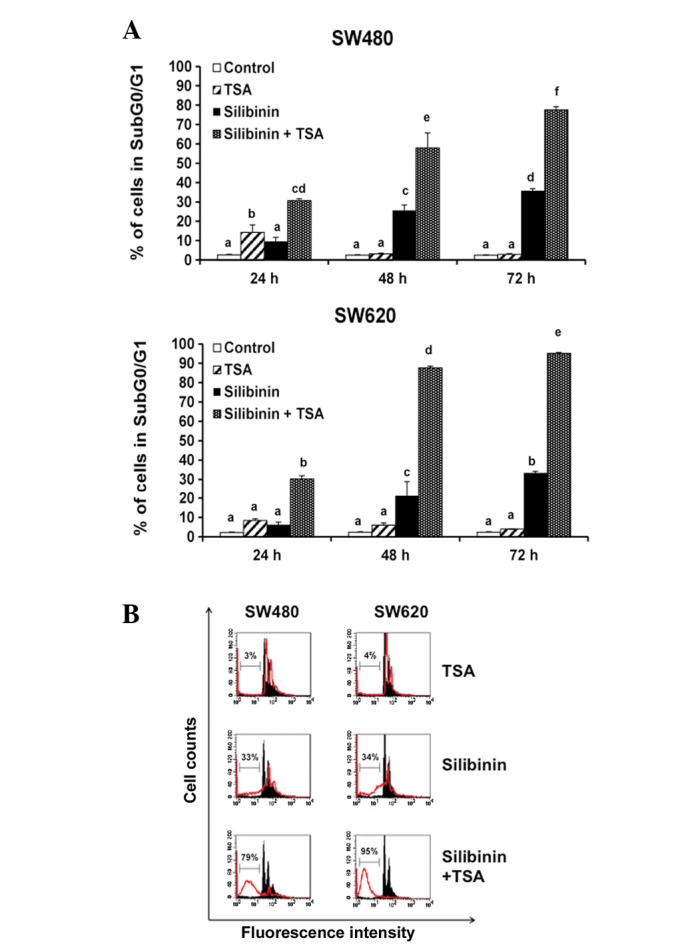
Cell death induced by silibinin and TSA. SW480 and SW620 cells were treated with DMSO 0.1% ± silibinin (300 *μ*M) ± TSA (0.1 *μ*M) for 24, 48 or 72 h. At each time point, SW480 and SW620 cells were harvested and stained with propidium iodide for the measurement of hypodiploid bodies and analyzed by flow cytometry as detailed in Materials and methods. In (A), data are presented as the mean ± standard error of three separate experiments. For each cell line, columns that do not share the same superscript differ significantly: ^*^P<0.05. (B) representative FACS histograms at 72 h; the black histogram represents the DMSO-only control cells while the red line represents the treated cells. The percentage of cells in the subG0/G1 region is indicated.

The clinically used HDAC inhibitor SAHA significantly enhanced silibinin-induced cell death (Fig. 2) but demonstrated no cell toxicity on its own. However, this effect was notably stronger in SW620 cells. To verify the interaction between silibinin and HDAC inhibitors, we used the broad spectrum HDAC inhibitor TSA and observed a clear synergistic effect with silibinin on cell death induction in both cell lines (Fig. 3). Cell death induction was more notable with TSA than with SAHA, and we found that the metastatic SW620 cells were more sensitive to the combined treatment than the adenocarcinoma SW480 cells.

## Discussion

Previously, we demonstrated that the polyphenol silibinin inhibited cell growth and induced apoptosis in SW480 and SW620 cells (21). In an azoxymethane-induced rat model, silibinin was able to prevent the formation of preneoplastic lesions, thus appearing to be a promising chemopreventive agent in CRC (22). Polyphenols have been shown to modify histone deacetylation and DNA hypermethylation, accompanying their chemopreventive effect (12–14,16,20).

In the present study, we investigated the epigenetic effects of silibinin. We found that silibinin did not change HDAC activity in SW480 and SW620 cells. These data contrasted with previous observations by Lah *et al* and Cui *et al* in hepatocarcinoma cells and xenografts (18,19), and by Mateen *et al* in non-small cell lung cancer (20), where silibinin was able to inhibit the activity of HDACs. However, silibinin reduced DNMT activity in both cell lines following 72 h of treatment. Inhibition of DNMT activity was already significant at 48 h in SW480 cells.

As other polyphenols and DNMT inhibitors have been demonstrated to act synergistically with HDAC inhibitors in cell death induction, we tested the effect of a combination of silibinin with two broad-spectrum HDAC inhibitors, SAHA and TSA, on the two cell lines. Both combinations synergistically induced cell death. These results were in agreement with those of other studies demonstrating that silibinin synergistically augmented the cytotoxic effects of SAHA and TSA in non-small cell lung cancer cells (20). Notably, in our study, SW480 and SW620 cells were both resistant to treatment by the HDAC inhibitors, which alone exhibited no cytotoxic effects.

However, the synergistic effect of silibinin and HDAC inhibitors could not be entirely attributed to silibinin-induced DNMT inhibition, as the increase in cell death occurred prior to significant DNMT inhibition by silibinin in SW620 cells. Furthermore, the synergy in cell death induction was stronger in SW620 cells (up to 95% cell death compared with 80% in SW480 cells), whereas DNMT inhibition was weaker in SW620 cells than in SW480 cells.

Silibinin and HDAC inhibitors possess pleiotropic anticancer activities, which may explain their synergistic effects; the ability of HDAC inhibitors to change the balance between pro- and anti-apoptotic factors (6) may contribute to the enhancement of the apoptosis-inducing properties of silibinin.

In conclusion, silibinin inhibited DNMT but not HDAC activity in colorectal SW480 and metastatic SW620 cells, and exerted synergistic effects with HDAC inhibitors on cancer cell death. Further investigations are required to determine the mechanisms involved in this process. However, our data suggest that treatments combining silibinin and HDAC inhibitors may represent a promising approach, given the non-toxic nature of silibinin and the fact that HDAC inhibitors selectively target cancer cells. Combined treatment of silibinin with different epigenetic agents, including HDAC inhibitors, in current clinical trials may thus contribute to the development of novel combination therapies.
